# Two New Phenolic Compounds from the Heartwood of *Caesalpinia sappan* L.

**DOI:** 10.3390/molecules19010001

**Published:** 2013-12-19

**Authors:** Ming-Bo Zhao, Jun Li, She-Po Shi, Chen-Qiu Cai, Peng-Fei Tu, Li Tang, Ke-Wu Zeng, Yong Jiang

**Affiliations:** 1 State Key Laboratory of Natural and Biomimetic Drugs, School of Pharmaceutical Sciences, Peking University Health Science Center, Beijing 100191, China; 2 Modern Research Center for Traditional Chinese Medicine, Beijing University of Chinese Medicine, Beijing 100029, China; 3 College of Life and Environmental Sciences, Minzu University of China, Beijing 100081, China

**Keywords:** *Caesalpinia sappan* L., dracaenone, epicaesalpin J, phenolic compounds

## Abstract

Two new phenolic compounds, epicaesalpin J and 7,10,11-trihydroxydracaenone, were isolated from the heartwood of *Caesalpinia sappan* L. Their structures were identified by spectroscopic analysis methods, such as 1D and 2D NMR, along with the high resolution mass spectral data. The NO inhibition activities of two new compounds and six known compounds were tested.

## 1. Introduction

**Figure 1:**
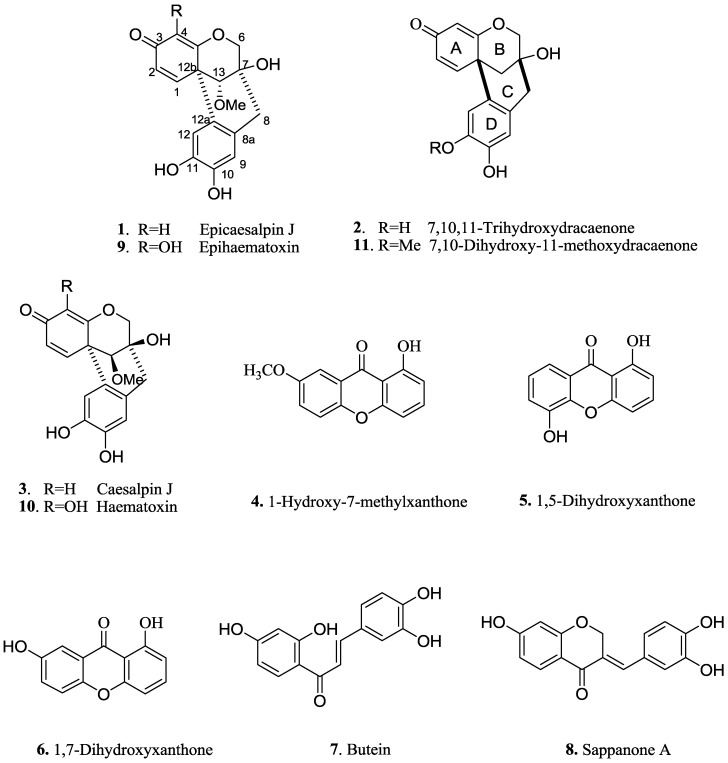
Structures of compounds **1**–**11**.

*Caesalpinia sappan* L. (Leguminosae) is widely distributed in Thailand, Indonesia, Vietnam, Burma, India and South and Southwest China [1]. The dried heartwood of this plant, Sappan Lignum, is popularly used as a Traditional Chinese Medicine for the treatment of menorrhalgia, cardiovascular and cerebrovascular diseases [2]. Previous investigations revealed that the extract of Sappan Lignum presented diverse and remarkable bioactivities, and therefore could be used as an anti-inflammatory, antimicrobial, antihypertensive and antiatherogenic agent. Prompted by the promising pharmaceutical properties, extensive studies on the phytochemical constituents of Sappan Ligunum have been carried out, which has resulted in the separation of various components including homoisoflavonoids [3,4,5,6,7,8,9], diterpenoids [10], dibenzoxocins [11,12,13,14,15,16,17,18], and a lactone [19]. As a part of our continuing studies on *C. sappan* [20,21], we report herein the isolation and structural identification of two new compounds **1** and **2** (Figure 1). Moreover, the NO inhibition activities of these new compounds and six known compounds **3**–**8** (Figure 1) were tested.

## 2. Results and Discussion

**Figure 2:**
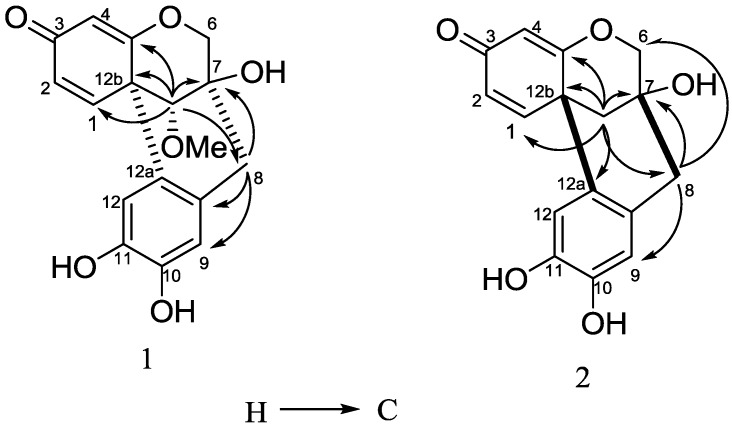
Key HMBC correlations of compound **1** and **2**.

**Figure 3:**
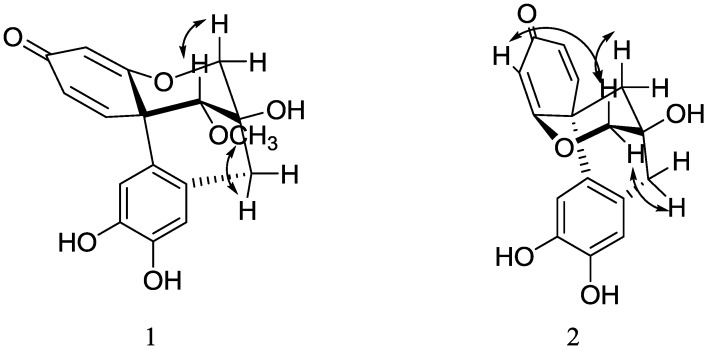
Key NOESY correlations of compounds **1** and **2**.

Compound **1** was obtained as a colorless gum, 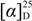 +317.3 (*c* 0.30, MeOH), UV λ_max_ (MeOH) nm: 212, 242. Its molecular formula was determined as C_17_H_16_O_6_ by HR-ESI-MS (found 317.1021 [M+H]^+^, calcd. 317.1025). The IR spectrum of **1** showed *α*,*β*-unsaturated ketone (1649 cm^−1^), aromatic ring (1595 cm^−1^) and hydroxyl group (3443 cm^−1^) absorptions. The ^1^H- and ^13^C-NMR spectra of **1** displayed the characteristic signals of a methoxyl group (*δ* 3.62), an oxymethylene (*δ* 4.18 and 3.79), an oxymethine (*δ* 3.48), an *α*,*β*-unsaturated ketone group, and a tetrasubstituted benzene ring, respectively. All protons and carbons were unambiguously assigned by 1D and 2D NMR experiments, including^ 1^H-^1^H COSY, HSQC, and HMBC (Table 1, Figure 2). Comparison of the NMR data with those of the known compound, caesalpin J (**3**) [11,21], revealed that **1** possessed a similar skeleton. The major difference is the upfield shift of H-13 from *δ* 3.84 in **3** to *δ* 3.48 in **1**, and the same phenomenon was also observed in the compounds haematoxin (**10**) and epihematoxin (**9**) [22], which suggested that **1** was a stereoisomer of **3**. The absolute configuration of **3** had been established in [12] by an X-ray crystallographic study of its triacetate. According to this paper, the ring B and C in **3** were both in a chair conformation. The optical rotation of **1** (+371.3) was consistent with that of **3** (+445.0), which suggested that **1** had the same configuration. In the NOESY spectrum of **1**, the cross peaks between H-13 and H-6*β*, and OMe-13 and H-8 unambiguously confirmed that **1** was a C-13 epimer of **3**, and the methoxyl group at C-13 was *α*-oriented (Figure 3). Thus, the structure of **1** was fully elucidated, and it was named epicaesalpin J.

**Table 1:** ^1^H-NMR (500 MHz) and ^13^C-NMR (125 MHz) data for 1 and 2 (*δ* in ppm and *J* in Hz).

No.	1 (in DMSO-*d*_6_)		1 (in CD_3_OD)		2 (in CD_3_OD)	
	*δ*_C_	*δ*_H_	*δ*_C_	*δ*_H_	*δ*_C_	*δ*_H_
1	146.3	7.03 d (10.0)	149.0	7.16 d (10.0)	151.5	6.88 d (10.0)
2	129.2	6.46 dd (10.0, 1.5)	130.4	6.51 dd (10.0, 1.5)	128.5	6.45 dd (10.0, 1.5)
3	187.1		190.9		191.4	
4	108.5	5.48 d (1.5)	109.8	5.57 d (1.5)	108.1	5.58 d (1.5)
4a	175.0		178.6		179.7	
6	77.8	4.12 d (11.0)	79.8	4.18 d (11.0)	81.0	3.88 dd (11.0)
		3.72 d (11.0)		3.79 d (11.0)		4.27 dd (11.0)
7	69.4		71.3		66.8	
8	37.6	3.16 d (16.0)	39.0	3.30 d (16.0)	43.5	3.16 d (16.0)
		2.83 d (16.0)		2.88 d (16.0)		3.10 d (16.0)
8a	122.6		124.2		129.5	
9	115.4	6.52 s	116.6	6.59 s	116.9	6.64 s
10	145.0		146.6		146.8	
11	143.6		145.2		145.1	
12	112.9	6.26 s	114.1	6.35 s	113.4	6.44 s
12a	126.6		128.5		125.5	
12b	51.1		53.5		47.8	
13	82.7	3.44 s	84.8	3.48 s	41.1	1.99 dd (11.5)
						2.34 dd (11.5)
OCH_3_	61.4	3.53 s	62.6	3.62 s	-	-

Compound **2** was obtained as a colorless gum, 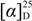 −152.6 (*c* 0.54, MeOH). Its molecular formula was determined as C_16_H_14_O_5_ by HR-ESI-MS (found 285.0765 [M−H]^+^, calcd. 285.0769). The ^1^H- and ^13^C-NMR data of **2** were similar to those of the known compound 7,10-dihydroxy-11-methoxydracaenone (**11**), except that **2** showed no methoxyl signals. Considering that the molecular weight of **2** was 30 Da less than that of **11**, compound **2** was identified as 7,10,11-trihydroxydracaenone. According to [23], the absolute configuration of **11** was different from those of **3** and **1**, and **11** had a C ring boat conformation. The optical rotation of **2** was −152.6, which was similar to that of **11** (-465.9). Moreover, in the NOESY spectrum, the cross peaks between H-4 and H-6*β*, and H-8 and H-6*α* unambiguously confirmed that **2** had the same configuration as **11**, which was opposite of that of **1** and **3** (Figure 3). All protons and carbons were unambiguously assigned by 1D and 2D NMR experiments, including ^1^H-^1^H COSY, NOESY, HSQC, and HMBC (Table 1, Figure 2 and Figure 3). Thus, the structure of **2** was confirmed as 7,10,11-trihydroxydracaenone.

**Scheme 1:**
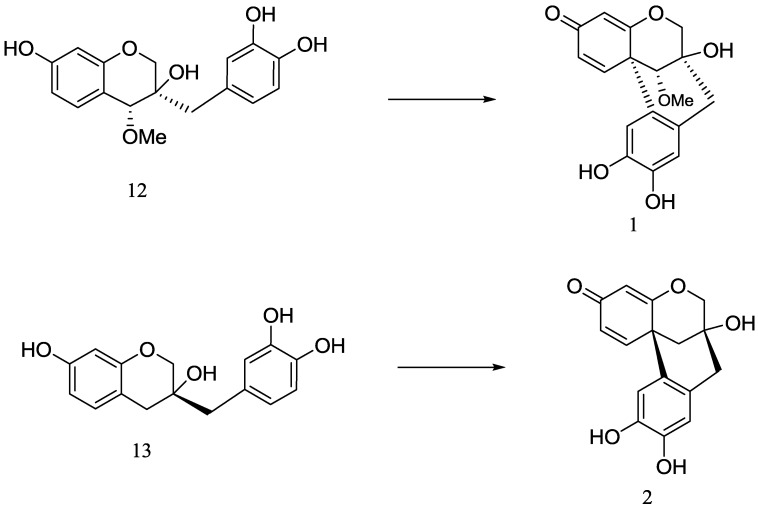
Proposed boigenetic pathway for **1** and **2**.

The dracaenone skeleton is uncommon in natural products, and only six compounds with this type of skeleton have been reported [11,22,23]. They are isolated from *Caesalpinia sappan* L. [11], *Haematoxylon campechianum* [22], and *Dracaena loureiri* Gagnep [23], although it is not unexpected that they exist in different genera, because it is believed that the dracaenone skeleton is the oxidative cyclization product of homoisoflavan in biogenetic pathway [24], so we deduced that **1** and **2** should be the oxidative products of **12** and **13**, respectively (Scheme 1).

Two new compounds and six known compounds caesalpin J (**3**), 1-hydroxy-7-methylxanthone (**4**), 1,5-dihydroxyxanthone (**5**), 1,7-dihydroxyxanthone (**6**), butein (**7**), and sappanone A (**8**) were evaluated for their inhibitory activities against nitric oxide production in LPS-activated BV-2 microglia according to a previously described method [25]. Compounds **4**, **6**, **7**, and **8** showed obvious inhibitory activity, with IC_50_ values that were lower than those of quercetin, the positive control (Table 2).

**Table 2:** Inhibitory effects of compounds **1**–**8** and quercetin.

Compound	Viability (at 50 µM, %)	IC_50_ (µM)	Compound	Viability (at 50 µM, %)	IC_50_(µM)
**1**	96.45	52.62	**6**	101.64	21.46
**2**	99.29	56.71	**7**	94.00	15.46
**3**	96.13	45.87	**8**	99.29	8.26
**4**	98.94	14.23	quercetin		23.42
**5**	101.19	28.65			

## 3. Experimental

### 3.1. General

Optical rotations were measured on an Autopol III automatic polarimeter (Rudolph Research Co., Hackettstown, NJ, USA). IR spectra were measured using a Thermo Nicolet Nexus 470 FT-IR spectrometer (Thermo Nicolet, Madison, WI, USA) with KBr disks. HR-ESI-MS were carried out on a Q-STAR ESI-TOF-MS/MS spectrometer (AB SCIEX, Framingham, MA, USA). 1D and 2D-NMR spectra were recorded on a Varian Inova-500 spectrometer (Varian, Palo Alto, CA, USA) with TMS as internal standard. Silica gel (200–300 mesh, Qingdao Marine Chemical, Qingdao, China) was used for column chromatography. Sephedex LH-20 gel was purchased from GE Healthcare Bio-Sciences AB (Uppsala, Sweden). MCI gel (CHP 20/P120) was purchased from Mitsubishi Chemical Industries Ltd. (Tokyo, Japan). Semi-preparative HPLC was performed on a Waters XBridge semi-preparative C-18 column (10 × 250 mm, 5 μm, Waters Co., Milford, CT, USA), eluting with MeOH/H_2_O at a flow rate of 2–3 mL/min; the detector used was DAD (200–400 nm) at room temperature. Fractions were monitored by TLC, and spots were visualized by spraying TLC plates with 10% sulfuric acid in ethanol and heating at 110 °C for 5–10 min.

### 3.2. Plant Material

Sappan Lignum (the heartwood of *Caesalpinia sappan* L.) was purchased from the Anguo medicinal materials market, Hebei Province of China, in September 2010. The plant material was authenticated by one of the authors, Prof. Peng-Fei Tu (School of Pharmaceutical Sciences, Peking University) and a voucher specimen (No.M-6-(5)) was deposited at the Herbarium of Peking University Modern Research Center for Traditional Chinese Medicine.

### 3.3. Extraction and Isolation

The dried heartwood of *Caesalpinia sappan* L. (21 kg) were chopped and extracted three times with 95% EtOH (168 L, 126 L, 126 L) to give 2.5 kg of crude extract. The extract was then suspended in water (5 L) and successively partitioned with petroleum ether, EtOAc and *n*-BuOH (20 L) to give after solvent removal fractions PE (60 g), EA (1,400 g), and BU (360 g), respectively.

A portion of EA (800 g) was subjected to silica gel column chromatography eluted with a step-wise gradient of CHCl_3_ and MeOH to obtain fractions 1–12. Fraction 5 was chromatographed on silica gel eluted with petroleum ether/EtOAc (3:1 to 1:1) to give subfractions 5a–f. Fraction 5e was separated by a silica gel column eluted with CHCl_3_/MeOH (25:1–10:1) to give 5ea–ed. Fraction 5ec was passed through Sephadex LH-20 eluted with CHCl_3_/MeOH (1:1), and further purified by semi-preparative HPLC (MeOH/H_2_O 85:15) to yield **1** (12.0 mg), along with **3** (10.0 mg). Fraction 8 was subjected to silica gel column chromatography eluted with CHCl_3_/Me_2_CO (10:1–1:1) to give 8a–h. Fraction 8d was subjected to a silica gel column eluted with CHCl_3_/MeOH (20:1–5:1) to give 8da–df. Fraction 8db was separated by a MCI column eluted with MeOH/H_2_O (30:70–100:0) to yield **2** (11.0 mg). The isolation of the known compounds had been reported in our previous papers [21,22].

### 3.4. Spectral Data

*Epicaesalpin J* (**1**). Colorless gum. 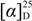 +371.3° (*c* 0.30, MeOH), UV λ_max_ (MeOH) nm: 212, 242. IR ν_max_ (KBr) cm^−1^: 3443, 2957, 1649, 1595, 1454, 1395, 1016. HR-ESI-MS *m/z*: 317.1021 [(M+H)^+^, calcd. for C_17_H_17_O_6_ 317.1025]. ^1^H-NMR and ^13^C-NMR (CD_3_OD and DMSO-*d*_6_) see Table 1.

*7,10,11-Trihydroxydracaenone* (**2**). Colorless gum. 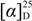 −152.6° (*c* 0.54, MeOH). UV λmax (MeOH) nm: **2**14, 240. IR ν_max_ (KBr) cm^−1^: 3381, 1653, 1591, 1522, 1451, 1395, 1065. HR-ESI-MS *m/z*: 285.0765 [(M−H)^−^, calcd. for C_16_H_14_O_5_ 285.0769]. ^1^H-NMR and ^13^C-NMR (CD_3_OD) see Table 1.

The spectral data of known compounds were reported in our previous literatures [20,21].

### 3.5. Inhibition of NO Production in LPS-Stimulated BV-2 Microglia

The assay was performed according to a previously described method [25]. Each compound was dissolved in DMSO and further diluted in the medium to produce different concentrations with a maximum concentration of 50 μM. The absorbance was measured at 570 nm with a Multilabel Plate Reader (Sunrise TECAN, Männedorf, Switzerland). Cytotoxicity was determined with the MTT assay. Quercetin (Sigma-Aldrich, Foster City, CA, USA) was used as the positive control.

## 4. Conclusions

The chemical study of the heartwood of *C. sappan* resulted in the isolation of two new compounds: epicaesalpin J (**1**) and 7,10,11-trihydroxydracaenone (**2**). Compounds **1** and **2** both have the dracaenone skeleton, which is uncommon in natural products. The new compounds, together with six known phenolic compounds, were evaluated for NO production inhibitory activity in LPS-stimulated BV-2 microglia. Compounds **4**, **6**, **7**, and **8** showed obvious inhibitory activity.
